# Accelerated Curing for Glass-Based Mortars Using Water at 80 °C

**DOI:** 10.3390/ma15062109

**Published:** 2022-03-13

**Authors:** Taohua Ye, Jianxin Lu, Zhenhua Duan, Lei Li, Dayu Zhu

**Affiliations:** 1 Department of Structural Engineering, College of Civil Engineering, Tongji University, Shanghai 200092, China; yeth@tongji.edu.cn (T.Y.); 2132319@tongji.edu.cn (L.L.); 2 Department of Civil and Environmental Engineering, The Hong Kong Polytechnic University, Hong Kong, China; jianxin.lu.lu@polyu.edu.hk; 3 Guangxi Key Laboratory of Disaster Prevention and Engineering Safety, Guangxi University, Nanning 530004, China

**Keywords:** glass aggregate mortar, accelerated curing, glass powder size, volume stability, compressive strength

## Abstract

The substitution of river sand with glass aggregate (GA) and cement with glass powder (GP) is a mainstream method to recycle waste glass. Traditionally, standard curing was widely used for glass-based mortars. However, it is time-consuming and cannot address low mechanical strengths of the early-age mortars. Therefore, the effect of water curing at 80 °C on the properties of GA mortars is investigated. Furthermore, the effect of the GP size is also considered. Results show that compared with the expansion of alkali-silica reaction (ASR), water curing at 80 °C has a negligible effect on the volume change. Moreover, the compressive strength of GA mortars under 1-day water curing at 80 °C is comparable with that under 28-day water curing at 20 °C. Therefore, the 1-day water curing at 80 °C is proposed as an accelerated curing method for GA mortars. On the other hand, the addition of GP with the mean size of 28.3 and 47.9 μm can effectively mitigate the ASR expansion of GA mortars. Compared with the size of 28.3 μm, GA mortars containing GP (47.9 μm) always obtain higher compressive strength. In particular, when applying the 1-day water curing at 80 °C, GA mortars containing GP (47.9 μm) can even gain higher strength than those containing fly ash.

## 1. Introduction

The amount of produced waste glass has been gradually surging with the increase of glass bottled consumer goods such as beer and wine. However, due to the fact that many cities do not have enough glass manufacturing industries, the recycling rate of waste glass is relatively low and a large amount of waste glass is urgent to be recycled. For example, in Hong Kong, about 106 kilotons of waste glass were disposed of at landfills in 2017 [1], while the recycling rate was only 12% [2]. On the other hand, there has been a consensus that the supply of river sand cannot satisfy the need for the production of mortars in the future due to its excessive exploitation [3]. In addition, the dramatic consumption would also bring about other environmental problems, such as the loss of river bed height and the instability of river course [3,4]. Therefore, for the purpose of efficiently recycling waste glass and effectively saving river sand, the substitution of river sand with waste glass as fine aggregates in mortars has been widely investigated [5,6,7].

In previous studies, the standard curing with the temperature of 20 ± 2 °C and the humidity of >95%, confirmed to the ISO standard, is the most commonly used curing method for glass aggregate (GA) mortars. However, this curing method is time-consuming and cannot address low mechanical strengths of the early-age mortars due to the use of GA. By contrast, various accelerated curing methods have been used in other types of mortars to improve performance. For example, Shi et al. reported that after the standard curing for 24 h followed by water curing at 80 °C for 48 h, alkali-activated slag mortars can obtain the highest compressive strength as the alkali-activation of the mortars was nearly completed [8,9]. However, the investigations about the application of water curing at high temperatures on GA mortars are still limited. It is well-known that the volume stability of GA mortars is one of the crucial issues that need to be considered because the amorphous SiO_2_ of GA can react with the alkalis of pore solution to form a kind of expansive product that would further damage the microstructure of the mortars [10,11,12]. This phenomenon is generally referred to as alkali-silica reaction (ASR). Therefore, limited investigations may be because the effects of water curing at high temperatures on the volume stability of GA mortars are unclear.

Traditionally, in order to mitigate the detrimental ASR expansion of GA mortars, supplementary cementitious materials (SCMs) are generally utilized, such as fly ash (FA) [11,13]. However, due to the consideration of carbon neutrality, the amount of coal usage is gradually decreased in China, leading to the decrease of FA production. Furthermore, there also exists a problem of uneven regional distribution for FA production [14]. Therefore, it is crucial to develop an alternative material to mitigate the ASR expansion of GA mortars. With the aim of recycling waste glass to a maximum extent, the utilization of glass powder (GP) would be a favorable choice. Currently, the effect of the GP addition on the properties of GA mortars under standard curing has been widely investigated [15,16,17]. Also, the effects of different GP sizes on the properties of the mortars under the standard curing have been reported in our previous studies [18,19,20,21]. However, the effects of the GP addition and the GP size on the properties of GA mortars under water curing at high temperatures are still unclear.

Based on the above discussion, the effects of water curing at high temperatures, the GP addition, and the GP size on the properties of GA mortars are investigated in this study. The volume stability and compressive strength of the mortars are mainly emphasized. According to the requirements of ASTM C1260, the ASR test is usually performed using the NaOH solution at 80 °C. Consequently, the particular temperature of 80 °C in this study was selected to not only investigate the effect of the water curing of high temperatures on the volume stability of the mortars but also distinguish the influence degree of high temperature (80 °C) and alkali environment (NaOH solution) on the volume stability of the mortars. In addition, the effects of different water-to-binder (w/b) ratios on the properties of GA mortars under accelerated curing are also analyzed. In summary, the aim of this study is to (1) reveal the feasibility of using water curing at 80 °C as an accelerated curing method for GA mortars; (2) illustrate the effects of the GP addition and the GP size on the properties of GA mortars under the accelerated curing and the superiority of GP having a suitable size compared with FA; and (3) extend the application range of the traditional accelerated curing method not only to shorten the curing age of glass-based mortars but also to effectively recycle waste glass.

## 2. Materials and Methods

### 2.1. Raw Materials

To achieve the aesthetic requirements of architectural mortars, a type of white ordinary Portland cement (OPC) from TAIHEIYO Cement Corp. (Tokyo, Japan) was used in this study. It contains 21.36 wt.% SiO_2_, 5.27 wt.% Al_2_O_3_, 67.49 wt.% CaO, 0.20 wt.% Fe_2_O_3_, and 0.048 wt.% Na_2_O. Furthermore, the GP was obtained after grinding GA by means of a laboratory ball mill (Deco Instrument Equipment Co., Ltd., Hunan, China), containing 73.50 wt.% SiO_2_, 0.73 wt.% Al_2_O_3_, 10.48 wt.% CaO, 0.38 wt.% Fe_2_O_3_, and 12.74 wt.% Na_2_O. In particular, after being milled for 0.5 h, 2 h, and 4 h, GP of different sizes could be produced and they were referred as to GP-0.5h, GP-2h, and GP-4h, respectively. In addition, the FA consisted of 45.70 wt.% SiO_2_, 19.55 wt.% Al_2_O_3_, 12.27 wt.% CaO, 11.72 wt.% Fe_2_O_3_, and 1.36 wt.% Na_2_O, derived from a local coal-fired power plant and generally produced as a byproduct during the generation of electricity.

**Figure 1:**
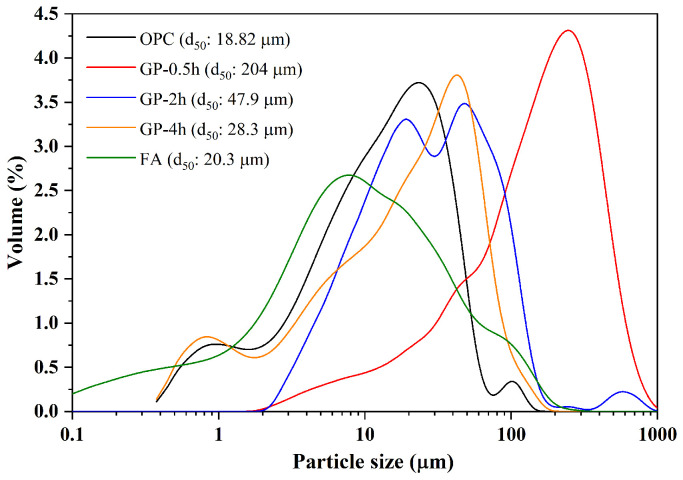
Particle size distributions of OPC, GP-0.5h, GP-2h, GP-4h, and FA.

**Figure 2:**
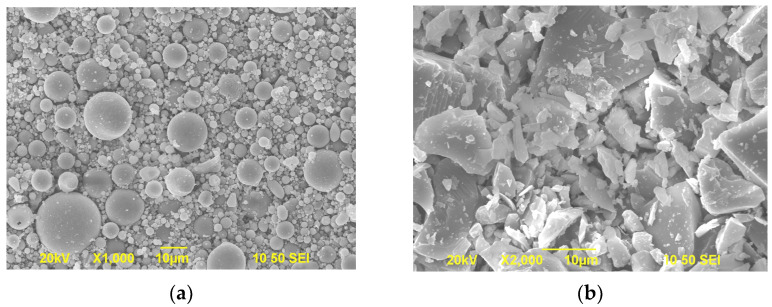
SEM images of GP-2h (**a**) and FA (**b**).

Figure 1 shows the particle size distributions of OPC, GP-0.5h, GP-2h, GP-4h, and FA. Moreover, the morphologies of GP-2h and FA were obtained using scanning electron microscopy (SEM) (Tescan, Brno, Czech), as shown in Figure 2. Obviously, FA consists of many spherical particles, while GP-2h exhibits an irregular shape with high aspect ratios and sharp edges.

**Figure 3:**
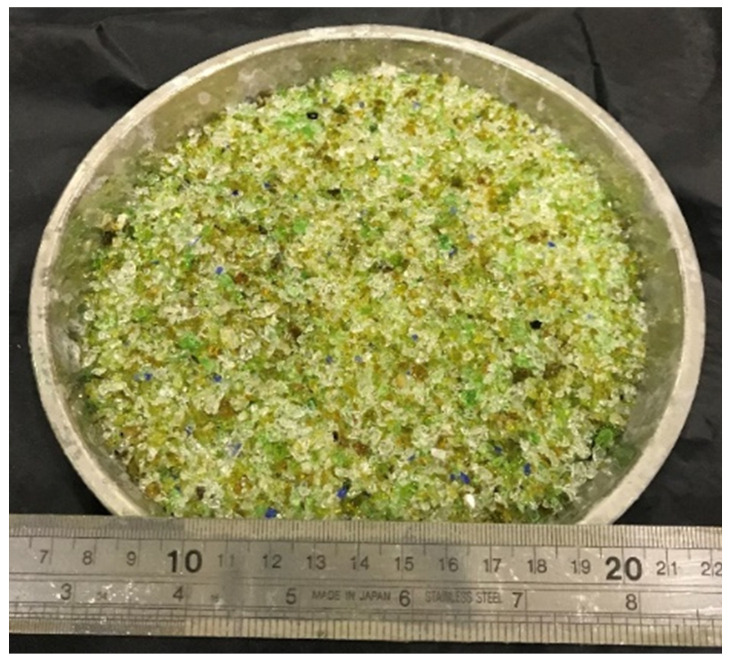
Appearance of GA.

**Figure 4:**
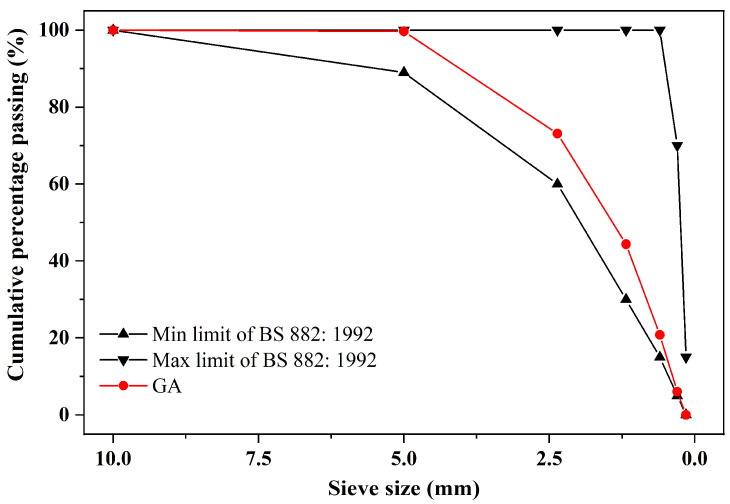
Particle size distribution of GA, conforming to the requirements of British standard BS 882:1992.

In Hong Kong, there are some manufacturing industries focusing on the recycling of waste glass, including postconsumer glass bottles. In this study, the provided GA was derived from waste beverage glass bottles. In particular, most of the waste glass was usually mixed with some contaminants such as paper. Therefore, before the preparation of mortars, GA was firstly washed with tap water, then oven-dried for 24 h at the temperature of 105 °C, and lastly sieved to achieve the dry state and also to screen out most of the contaminants. The appearance and particle size distribution of GA are presented in Figure 3 and Figure 4, respectively.

### 2.2. Test Methods

#### 2.2.1. Sample Preparation

Table 1 lists the mixing proportions of different mixtures. In series 1, the w/b ratio of 0.41 and the SCMs content of 20 wt.% were used. In this series, the reference sample and the samples containing GP-0.5h, GP-2h, GP-4h, and FA are referred to as Control 0.41, GP 0.5, GP 2, GP 4, and FA 20, respectively. Furthermore, the effect of different w/b ratios on the properties of GA mortars was investigated in series 2. In this series, the same naming rules are used.

The synthesis of the mortars proceeded as follows. Firstly, the solid materials (binder + aggregate) were mixed for 1 min. Subsequently, water was added to the solid materials. Then, the mixtures were stirred at low rotation speed (62 ± 5 r/min) for 2 min, followed by stirring at high rotation speed (125 ± 10 r/min) for another 2 min. Finally, the obtained samples were cast into steel molds. Each mold was put on a vibrating table for 15 s for compaction.

Meanwhile, the consistency of the fresh mortars was tested based on the requirements of the Chinese standard JGJ70-2009. It can be found in Table 1 that with the addition of FA and GP-0.5 h, the consistency of GA mortars would increase, while with the decrease of the particle size of GP, the consistency of GA mortars would begin to decrease. Furthermore, it was also observed that with the increase of the w/b ratio, the consistency of GA mortars could be significantly improved.

#### 2.2.2. Volume Stability Test

Alkali curing at 80 °C—According to ASTM C1260, the volume change of the samples under alkali curing at 80 °C can be referred to as the ASR expansion. Therefore, this change was determined by the procedures described in ASTM C1260. The tested samples with a dimension of 25 × 25 × 285 mm^3^ were prepared according to the above synthesis route. After demolding, the length of three tested samples from each group was recorded as the initial length. And then, these samples were immersed in sealed containers filled with deionized water and then stored in a vacuum oven at 80 °C for 1 day to record as the zero point. After that, the samples were turned to other sealed containers containing 1 mol/L NaOH solution, and still stored in the oven at 80 °C. Subsequent changes in the sample lengths were monitored after the immersion of 1, 3, 7, 14, 21, and 28 days. Finally, the average measurement from three samples was reported as the expansion value of this group.

Water curing at 80 °C—The volume change of the samples under water curing at 80 °C was tested and recorded with the same method as above. Similarly, three samples from each group were needed for the test. Note that the samples under the water curing were always immersed in the water and the NaOH solution (Sinopharm Chemical Reagent Co. Ltd., Shanghai, China) was not applied.

Water curing at 20 °C—The volume change of three samples of group Control 0.47 under water curing at 20 °C was also tested and recorded with the same method.

#### 2.2.3. Compressive Strength Test

For comparing the effects of water curing at 80 °C and alkali curing at 80 °C on the compressive strength of GA mortars, the samples with a dimension of 40 × 40 × 40 mm^3^ were cured under environments similar to those used by the volume stability test. While performing the volume stability test, three corresponding cubic samples of each group were selected and their compressive strengths were tested using a compaction machine (Luda Mechanical Instrument Co., Ltd., Zhejiang, China) with a maximum capacity of 300 kN. The load was increased at a rate of 0.6 MPa/s until failure, which conformed to the requirements of the Chinese standard GB/T 17671-1999. In addition, the compressive strength of three samples of group Control 0.41 under water curing at 20 °C was also tested. The test ages were 7, 28, and 90 days, respectively.

Note that although the samples with different sizes (e.g., 25 × 25 × 285 mm^3^ and 40 × 40 × 40 mm^3^) may indeed have had different degrees of hydration under the same curing environment, it did not affect the main conclusions of this study. On the one hand, the size of the samples used in this study was determined according to the requirements of the above standards (i.e., ASTM C1260 and GB/T 17671-1999). On the other hand, because the size of the samples in each experiment was consistent, i.e., the size of the samples was not a variable in this study, the main conclusion of this study cannot be affected by the size of the samples.

#### 2.2.4. Scanning Electron Microscope (SEM) Test

After the compressive strength test, fragments from the crushed samples were collected and soaked into anhydrous ethanol for one week, followed by oven-drying at 60 °C to remove the residual ethanol. Finally, their morphological observations were performed by using the SEM test (Tescan, Brno, Czech).

## 3. Results and Discussion

### 3.1. Feasibility of the Water Curing at 80 °C on GA Mortars

#### 3.1.1. Volume Change Analysis

**Figure 5:**
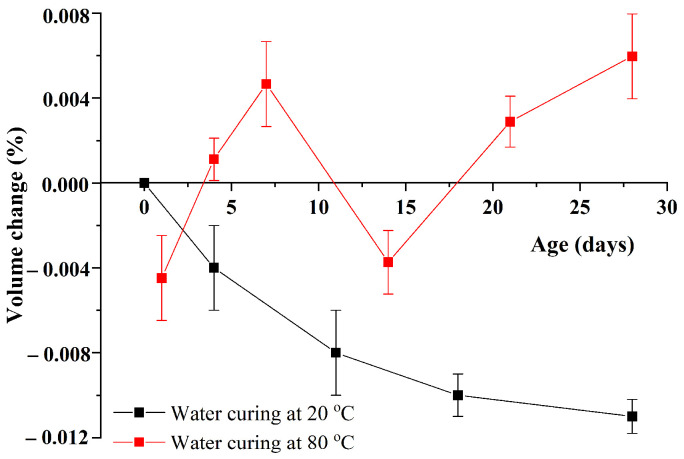
Volume changes of the sample Control 0.41 under water curing at 20 °C and 80 °C.

Figure 5 compares the volume changes of the sample Control 0.41 under water curing at 20 °C and 80 °C. It can be obviously seen that like the plain mortars, GA mortars under curing at 20 °C shrink. Furthermore, as the reaction goes on, the shrinkage rate of GA mortars gradually slows down. Generally, the former one is attributed to the volume differences between reactants (clinker + water) and hydration products during cement hydration. On the other hand, the latter one owes to the reduction in the reaction rate of cement hydration.

It can be also found from Figure 5 that the volume change of the sample Control 0.41 under curing at 80 °C is apparently different from that under curing at 20 °C, indicating that curing at elevated temperatures may have a remarkable effect on the volume change of GA mortars. Moreover, it was found that the volume change of the sample Control 0.41 under curing at 80 °C always fluctuated around the zero point, of which the values ranged from −0.005% to 0.006%. According to previous studies [22,23,24], curing at elevated temperatures can significantly increase the mobility of ions. Therefore, curing at 80 °C cannot only accelerate the cement hydration [25] but also facilitate the ASR expansion [26]. Consequently, different from curing at 20 °C where the cement hydration controls the volume change of GA mortars, the volume change of GA mortars under curing at 80 °C depends on the synergetic effect of both hydration shrinkage and ASR expansion. More investigations on the explanations of this phenomenon are needed.

**Figure 6:**
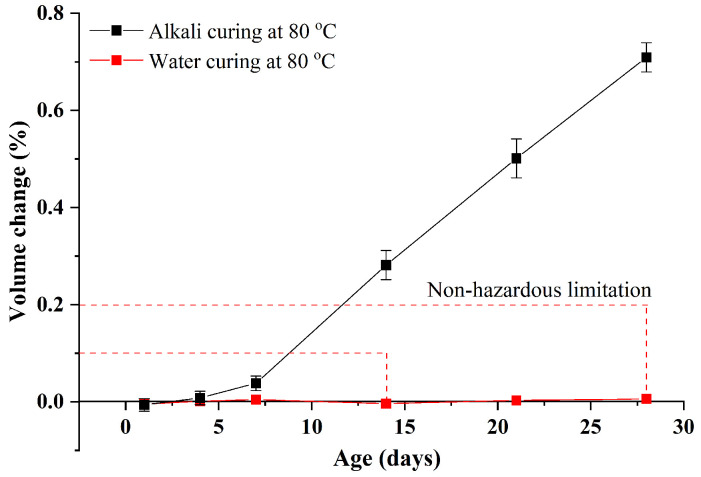
Volume changes of the sample Control 0.41 under alkali curing at 80 °C and water curing at 80 °C.

To further illustrate the influence of water curing at 80 °C, the volume changes of the sample Control 0.41 under alkali curing at 80 °C and water curing at 80 °C are also compared, as exhibited in Figure 6. It can be clearly seen that compared to alkali curing at 80 °C (the standard test of accelerated ASR conformed with ASTM C1260), the influence of water curing at 80 °C is negligible, even though the volume change of GA mortars under water curing at 80 °C is significantly different from that under water curing at 20 °C. This phenomenon indicates that compared to curing at elevated temperatures, a highly alkaline environment plays a more decisive role in the alkali expansion of GA mortars, which can refer to alkali-activated concrete that its ASR expansion is usually larger than that of corresponding ordinary concrete, due to a huge amount of the Na_2_O addition [27].

In addition, it should be noted that under alkali curing at 80 °C, the ASR expansion of the sample Control 0.41 is very large. More specifically, the ASR expansion values of the sample under alkali curing at 80 °C at 14 and 28 days are around 0.28% and 0.71%, respectively, which are far beyond the limits required by ASTM C1260, as shown in Figure 6. It confirms a widely held view that when the GA is used, the ASR expansion of cementitious materials should be particularly emphasized and solved.

#### 3.1.2. Compressive Strength Analysis

**Figure 7:**
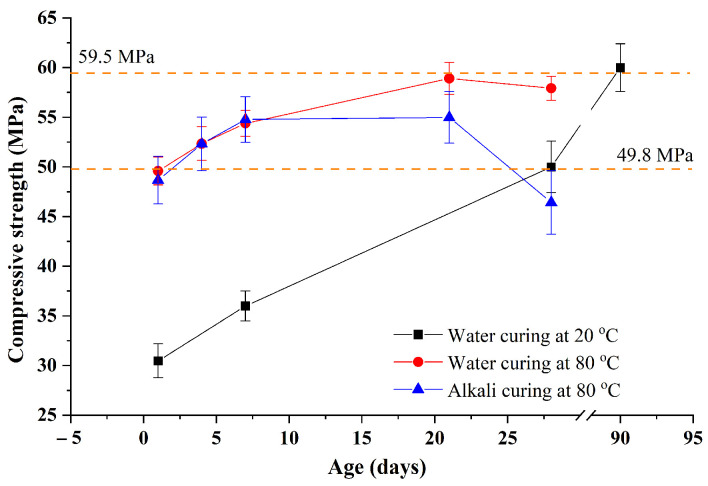
Compressive strengths of the sample Control 0.41 under different conditions.

After clarifying the influence of water curing at 80 °C on the volume change of GA mortars, the compressive strengths of the sample Control 0.41 under different conditions are shown in Figure 7 to illustrate the effect of water curing at 80 °C on the strength of GA mortars. Firstly, it can be clearly seen that when the hydration age prolongs from 1 to 90 day, the compressive strength of GA mortars under water curing at 20 °C gradually increases from 30.47 to about 60 MPa. This increment can be attributed to the continuous reaction of cement hydration, resulting in the formation of a denser microstructure.

Furthermore, it can be also obviously concluded from Figure 7 that compared to water curing at 20 °C, GA mortars under water curing at 80 °C can rapidly obtain the higher compressive strength in most cases. For example, when 7-day curing is used, the compressive strength of GA mortars increases from about 36 to 54.39 MPa with the increase of curing temperature of 20–80 °C. According to Silva et al. [23], elevated temperatures can lead to the increase of transportation and reaction rates of ions. Consequently, due to the higher reaction rate, curing at 80 °C can endow the mortars with more superior compressive strength.

It should be noted that under water curing at 80 °C, the compressive strength of GA mortars can firstly increase and then decrease with the increase of the curing age. More specifically, when the age increases from 1 to 21 day, the strength of GA mortars increases from 49.59 to 58.92 MPa due to the continuous reaction of cement hydration. However, when the age further increases to 28 day, the strength slightly decreases to 57.93 MPa. This is extremely different from the results reported by previous studies where water curing at elevated temperatures was used [28,29]. Generally, under water curing at elevated temperatures, the compressive strength of Portland cement mortars or concrete can be increased followed by being constant. This is because the cement hydration reaction can proceed quickly under water curing at elevated temperatures, therefore the strength showing a trend of rapid growth; then after a certain period of curing, the cement hydration is almost completed, thus the strength further exhibits a stable trend. On the other hand, this decrement cannot be also explained by the results reported by previous studies where steam curing was applied. According to these studies, the decrease in strength can be owing to the rapid water loss of the samples [23,30]. It can be also due to the contraction of C-S-H gels due to excessive shrinkage and dehydration [31], consequently inducing cracks. However, the water loss of mortars is an uncommon phenomenon during water curing. Therefore, it is crucial to explain the decrease of the compressive strength of GA mortars under water curing at 80 °C. As shown in Section 3.1.1, the volume change of GA mortars under water curing at 80 °C is controlled by the synergetic effect of both hydration shrinkage and ASR expansion. Here, this synergetic effect is also used to explain the development of the compressive strength of GA mortars under water curing at 80 °C. In other words, the cement hydration is almost completed after a certain period of curing, while the ASR expansion is still developing. Consequently, the loss of the compressive strength of GA mortars under water curing at 80 °C can be attributed to the damage of the microstructure derived from the growing and swelling of ASR gels. It is consistent with the trend that after the 17-day water curing at 80 °C, GA mortars are continuously expanding, as shown in Figure 5.

In particular, with the help of high-temperature curing, the 1-day and 21-day compressive strengths of GA mortars are similar to their 28-day and 90-day compressive strengths under water curing at 20 °C, which are about 49.8 and 59.5 MPa, respectively. Therefore, combined with the analyses of volume change and compressive strength tests, it indicates that water curing at 80 °C can be used as a kind of nonhazardous accelerated curing method to effectively shorten the curing duration of GA mortars and facilitate the preparation or construction of the mortars. Considering that the peak strength of GA mortars under water curing at 80 °C appears in the range of 7–28 days and the strength reduction after the peak is always associated with the deterioration of microstructure, the 1-day water curing at 80 °C is suggested to use for GA mortars.

**Figure 8:**
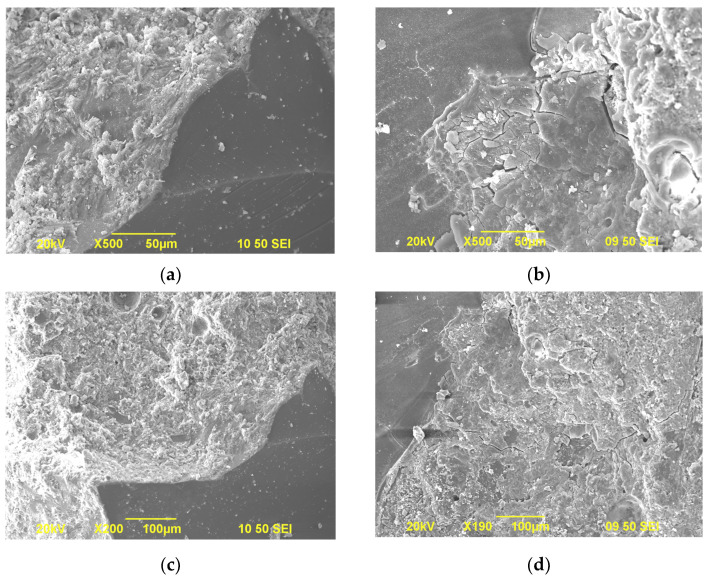
SEM images of Control 0.41 under the 7-day water curing at 80 °C (**a**,**c**) and the 7-day alkali curing at 80 °C (**b**,**d**).

Finally, it can be found from Figure 7 that, under alkali curing at 80 °C, the compressive strength of GA mortars also increases and then decreases with an increase in the curing age. Similarly, the increment can be attributed to the promotion of cement hydration and the decrement can be owing to the continuous development of ASR expansion. It can be also observed that compared to water curing at 80 °C, the compressive strength of GA mortars under alkali curing at 80 °C is subequal within the 7-day curing and then significantly lower after that. This phenomenon can be explained by the changes of their volume. As shown in Figure 6, within the 7-day curing, the volume change of GA mortars under alkali curing at 80 °C is close to that under water curing at 80 °C, while beyond the 7-day curing the change of the mortars under alkali curing at 80 °C is significantly higher than that under water curing at 80 °C. Therefore, this remarkable reduction in the compressive strength of GA mortars after the 7-day alkali curing at 80 °C can be attributed to the enormous expansion of ASR gels, leading to the severe damage of the microstructure. To confirm this speculation, the microstructure of GA mortars under different curing regimes is observed by SEM tests, as shown in Figure 8. It was found that after the 7-day curing, the microstructure of GA mortars under water curing at 80 °C is almost not damaged and there are no obvious cracks and pores found in the interfacial transition zone (ITZ) between the GA and the paste. On the contrary, an eroded microstructure could be noticed in GA mortars after the 7-day alkali curing at 80 °C, along with a great number of cracks and pores in the ITZ, which is derived from the swelling ASR gels.

#### 3.1.3. Effect of w/b Ratios

**Figure 9:**
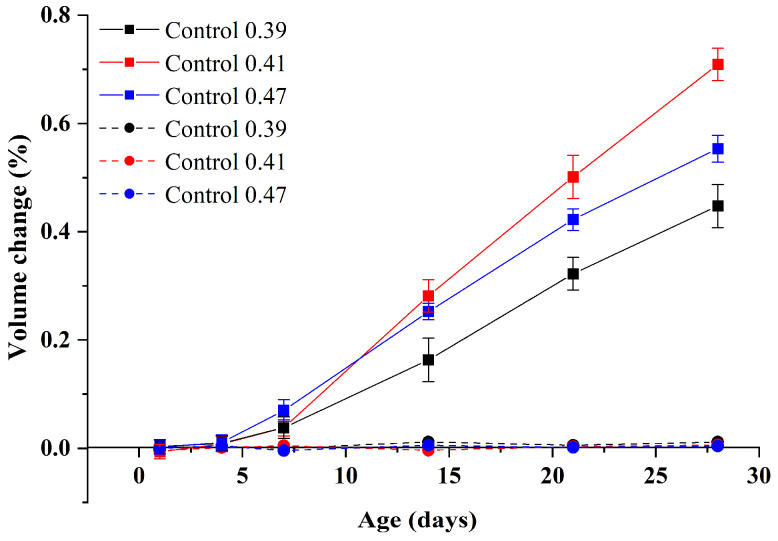
Volume changes of GA mortars with different w/b ratios under alkali curing at 80 °C (solid line) and water curing at 80 °C (dash line).

**Figure 10:**
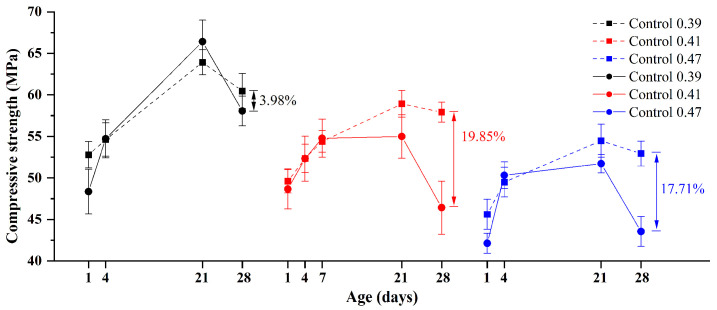
Compressive strengths of GA mortars with different w/b ratios under alkali curing at 80 °C (solid line) and water curing at 80 °C (dash line).

The influence of different w/b ratios on the properties of GA mortars is also investigated. Figure 9 and Figure 10 show the volume change and compressive strength of Control 0.39, Control 0.41, and Control 0.47 under alkali curing at 80 °C and water curing at 80 °C. As expected, under water curing at 80 °C, the influence of different w/b ratios on the volume change of GA mortars is negligible. However, the volume change of GA mortars under alkali curing at 80 °C exhibits an extremely different trend. It can be found that when the w/b ratio is decreased from 0.47 to 0.39, the ASR expansion of GA mortars would be increased and then decreased. It may suggest that when considering ASTM C1260 that requires the targeted material with the w/b ratio of 0.47, its ASR expansion, especially the 28-day one, may be underestimated.

According to Hu et al. [32], although a lower w/b ratio can result in a higher heat of hydration rate at early hours, the total heat of hydration of mortars with different w/b ratios within the first 24 h was found to be approximately the same. Therefore, the volume change of GA mortars with different w/b ratios may be not controlled by the hydration degree. However, as shown in Figure 10, the compressive strength of GA mortars under water curing at 80 °C still decreases with the increase of w/b ratios. Generally, it can be attributed to the excess of water being evaporated by the heat of hydration before demolding, which results in the formation of more pores and shrinkage cracks in the mortars containing a huge amount of water [33,34]. Based on these explanations, compared to the sample Control 0.41, the lower ASR expansion of the sample Control 0.47 can be due to the fact that a large number of pores provide adequate room for accommodating the swelling ASR gel. On the other hand, the lower ASR expansion of the sample Control 0.39 can be owing to a denser microstructure, which endows the mortars with the higher resistance toward the expansive stress derived from the swelling ASR gel. Consequently, with the increase of w/b ratios, the ASR expansion of GA mortars under alkali curing at 80 °C can increase and then decrease, which is consistent with the strength loss of GA mortars, as shown in Figure 10.

### 3.2. Effects of GP Addition and Fineness on GA Mortars under 80 °C Curing

#### 3.2.1. Volume Change Analysis

**Figure 11:**
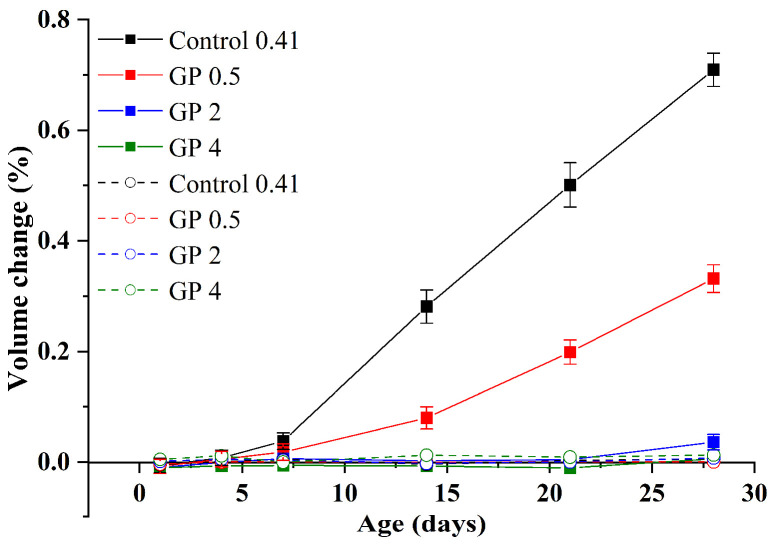
Volume changes of GA mortars under alkali curing at 80 °C (solid line) and water curing at 80 °C (dashed line).

According to the analysis of Section 3.1, water curing at 80 °C can be used as a nonhazardous accelerated curing method on GA mortars, while the enormous ASR expansion of the mortars is still an urgent problem. Figure 11 shows the influence of GP addition and fineness on the volume changes of GA mortars under alkali curing at 80 °C and water curing at 80 °C. Similar to GA mortars without the GP addition, the volume changes of GA mortars containing different GP sizes under water curing at 80 °C also fluctuate at zero point, of which the values are ranging from −0.005% to 0.012%. These tiny values indicate that both GP addition and fineness have no obvious effect on the volume change of GA mortars under water curing at 80 °C.

It can be also clearly observed from Figure 11 that GP addition can significantly mitigate the ASR expansion of GA mortars under alkali curing at 80 °C. More specifically, no matter what the mean particle size of GP is, the ASR expansion of the GA mortars with GP addition is reduced from 0.28% to below 0.1% under the 14-day alkali curing at 80 °C. It is well known that FA can effectively mitigate the ASR expansion of GA mortars due to several reasons as follows. Firstly, FA addition can reduce the alkalinity of the cement pore solution due to the dilution effect and the reduction on ion diffusion coefficient [35,36]. Then, FA addition can produce C-S-H gels with low Ca/Si ratios which have a higher capacity to absorb a higher quantity of alkalis than those with high Ca/Si ratios [37]. Furthermore, FA addition can reduce the permeability of cementitious materials to hinder the migration of alkalis to GA [38]. Finally, FA addition can improve the strength for providing higher resistance to the expansive stress from the swelling ASR gel [36]. Here, because of similar chemical compositions of GP and FA, this phenomenon can be explained by several mitigation mechanisms of FA toward ASR expansion of GA mortars, such as the dilution effect and the formation of C-S-H gels with low Ca/Si ratios. However, differently from FA, the GP addition cannot reduce the permeability and improve the strength of GA mortars. According to Raju et al. [39], when the 5 wt.% GP was added, the chloride penetration and water permeability of GP concrete were significantly improved, while the 20 wt.% GP addition almost no changed the performance of GP concrete. Also, according to the strength analysis below, we found that with the addition of 20 wt.% GP, the compressive strength of GA mortars is significantly decreased. Therefore, in addition to the dilution effect and the formation of C-S-H gels with low Ca/Si ratios, the remarkable mitigation effect of the GP addition on the ASR expansion of GA mortars should be particularly explained. On the one hand, less calcium phase from GP can mitigate the ASR expansion of GA mortars due to the increase of formation time of the ASR gel. A similar phenomenon has been reported in conventional concrete [40]. On the other hand, although GP contains a large number of alkalis, it can consume more alkalis (e.g., calcium hydroxide in pore solution) to form the sodium calcium silicate hydrate gel owing to the pozzolanic reaction [21], which reduces the mobility and availability of alkalis, leading to lower expansion around GA. Moreover, our previous study [18] exhibited that the GP addition can improve the ITZ in mortars. Therefore, it is believed that the GP addition can improve ITZs between GA and matrix, further reducing the penetration of aggressive alkalis and the dissolution of silicate ions.

It should be noted that although the ASR expansion of GP 0.5 under the 14-day alkali curing at 80 °C is satisfactory, its expansion (~0.33%) under the 28-day alkali curing at 80 °C is still far beyond the limitation (0.2%) of ASTM C1260. This can be attributed to the unexpected trend that, as the alkali curing proceeds, the ASR expansion of GA mortars firstly exhibits a moderate increase within 7-day curing and then dramatically surges after 7-day curing, as shown in Figure 11. Under this circumstance, the ASR expansion of GA mortars is usually underestimated, since the existing studies were generally concerned about their 14-day expansion and neglected their 28-day expansion. In fact, this trend is not a common one for the ASR expansion of concrete. For example, Long et al. [41] indicated that the ASR expansion of alkali-activated glass mortars always exhibited an accelerated growth and then a steady state. Here, this unexpected trend can be explained as follows. Due to the robustness of the microstructure, the growing and swelling of initially formed ASR gels are restricted. At the same time, the ITZ becomes denser due to the appearance of ASR gels, resulting in the increase of strength at the early ages (Figure 7) and the inner glass being covered. Therefore, the initial development of ASR expansion of GA mortars is relatively slow. It is worth noting that since the matrix has reached a high hydration degree/compressive strength under alkali curing at 80 °C in the first few days (Figure 7), the hydration process is very slow in the subsequent curing. By contrast, ASR gels are still constantly growing and swelling. When the expansive stress of ASR gels is greater than the resistance of the microstructure, cracks would be occurred. Moreover, with the proceeding of the expansion, the microstructure would gradually collapse and the ITZ would become loose again, allowing the inner glass to be in contact with the outer alkaline pore solution. Consequently, it would lead to the rapid growth of the ASR expansion of GA mortars and the significant reduction of their compressive strength (Figure 7).

Based on the above analysis, it suggests that GP-0.5 h cannot be used to mitigate the ASR expansion of GA mortars, while the addition of finer GP may be the better choice. As shown in Figure 11, within the 28-day alkali curing at 80 °C, the volume changes of GP 2 and GP 4 are always below 0.036%, indicating that the addition of finer GP can effectively mitigate the ASR expansion of GA mortars. According to Ichikawa [42], this can owe to the particle size of GP less than 50 μm (large specific surface area), which would preferentially react with alkali hydroxide to prevent the formation of expansive pressure after the formation of tight reaction rims.

#### 3.2.2. Compressive Strength Analysis

**Figure 12:**
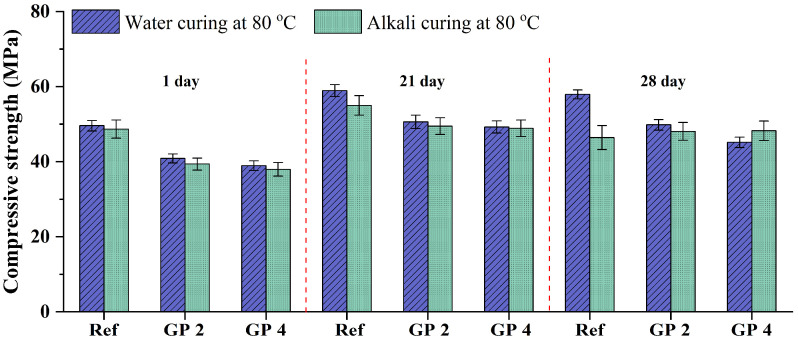
Compressive strengths of GA mortars under alkali curing at 80 °C and water curing at 80 °C.

The influence of the addition of GP-2h and GP-4h on the compressive strengths of GA mortars under 28-day alkali curing at 80 °C and 28-day water curing at 80 °C is shown in Figure 12. In particular, since the compressive strengths of GA mortars under 1-day and 21-day water curing at 80 °C are respectively comparable with those under 21-day and 90-day water curing at 20 °C, the 1-day and 21-day compressive strengths of GP 2 and GP 4 mortars under alkali curing at 80 °C and water curing at 80 °C are also exhibited.

As shown in Figure 12, compared to the 1-day and 28-day curing, GA, GP 2, and GP 4 mortars under the 21-day curing obtained the highest compressive strengths, which is consistent with the analysis of Section 3.1.2. The increments can be attributed to the continuous proceeding of cement hydration, while the decrements can be owing to the deterioration of the microstructure derived from the swelling of the ASR gel.

It can be also found from Figure 12 that the compressive strength of GA mortars is always decreased with the addition of GP-2h and GP-4h in most cases. It can be attributed to the low pozzolanic activity of GP. Furthermore, compared to GA and GP 2 mortars, GP 4 mortars always have the lowest compressive strength. For example, the compressive strengths of GA, GP 2, and GP 4 mortars under the 1-day water curing at 80 °C are 49.59, 40.86, and 38.94 MPa, respectively. According to Afshinnia and Rangaraju [43], the pozzolanic effect of the finer GP was likely to be sufficient to overcome the dilution effect. Lu et al. [18] further indicated that the finer GP with a larger specific surface area can facilitate the pozzolanic effect. Lu et al. [19] also confirmed these views through the test of hydration heat and reported that with the increase of the specific surface area of GP, the hydration degree of GP mortars can be improved and consequently the microstructure becomes denser. Based on these explanations, the compressive strength of GP 4 mortars should be higher than that of GP 2 mortars; however, it is completely different from the trend reported in this study. Here, we suggest that the higher compressive strength of GP 2 mortars may derive from the synergetic effects of hydration degree and particle packing density. In other words, although the hydration degree of GP 2 mortars is slightly lower than that of GP 4 mortars, GP 2 mortars may have a larger particle packing density. As shown in Figure 1, GP-4h has a much similar particle size distribution with OPC, which would lead to the loss of the interparticle filling effect, further resulting in the formation of a large amount of the intergranular pores. Therefore, compared to 20 wt.% GP-4h, the addition of 20 wt.% GP-2h can not only effectively mitigate the ASR expansion of GA mortars, but also provide more superior strength under water curing at 80 °C.

In addition, it can be obviously seen from Figure 12 that differently from GA mortars, the compressive strengths of GP 2 and GP 4 mortars under alkali curing at 80 °C are always similar to those under water curing at 80 °C. According to the analysis of Section 3.2.1, the influence of the ASR expansion on the volume change of GP 2 and GP 4 mortars is negligible. Therefore, similarly to water curing at 80 °C, the change in their compressive strengths under alkali curing at 80 °C is also mainly affected by the elevated temperatures, even though that effect is also weak (Section 3.1.1). Consequently, under the effect of the same factor, the limited differences in the change of compressive strength can be detected.

### 3.3. A Comparison of GP and FA

**Figure 13:**
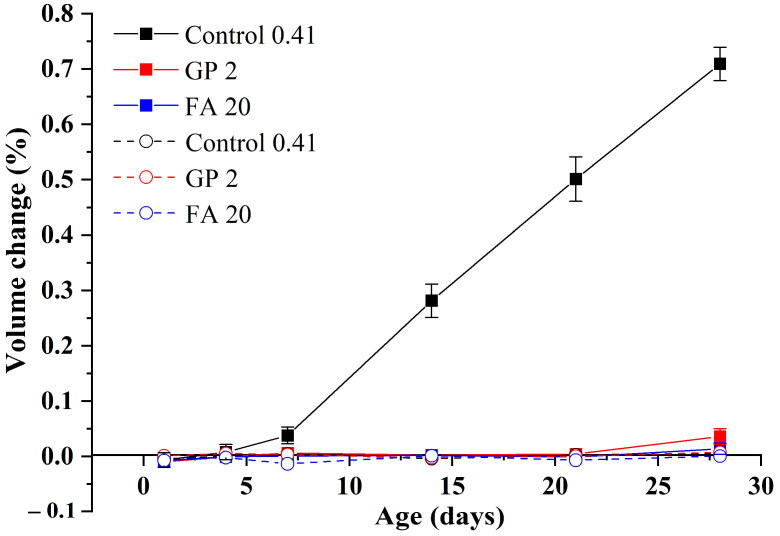
Volume changes of GP 2 and FA 20 mortars under alkali curing at 80 °C (solid line) and water curing at 80 °C (dash line).

According to the analysis of Section 3.2, GP-2h may be an alternative material to substitute FA in GA mortars under water curing at 80 °C. For further illustrating the advantage of GP-2h, the influence of the addition of GP-2h and FA on the volume change of GA mortars is shown in Figure 13. Obviously, there are similar volume changes of GP 2 and FA 20 mortars under both alkali curing at 80 °C and water curing at 80 °C, which are much lower than the change of GA mortars under alkali curing at 80 °C. It indicates that when considering the volume change of GA mortars, GP-2h can obtain comparable effects with FA.

**Figure 14:**
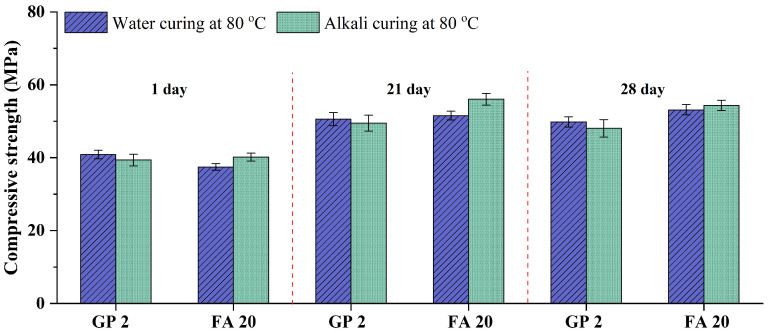
Compressive strengths of GP 2 and FA 20 mortars under alkali curing at 80 °C and water curing at 80 °C.

Figure 14 also shows the influence of the addition of GP-2h and FA on the compressive strength of GA mortars. It can be clearly seen that compared to FA 20 mortars, the compressive strength of GP 2 mortars under water curing at 80 °C is always lower, except the 1-day one. For example, the compressive strengths of GP2 and FA 20 mortars under the 1-day water curing at 80 °C are 40.86 and 37.44 MPa, respectively. This can be attributed to the higher pozzolanic effect of GP-2h at the early age. It is consistent with the phenomenon reported by previous studies that when adding FA, cementitious materials generally need the curing of a long time to gain satisfactory mechanical properties [44,45]. Therefore, according to the suggestion of Section 3.1, when the 1-day water curing at 80 °C is used an accelerated curing method, the addition of GP-2h is the better choice.

In particular, different from GP 2 mortars, the compressive strength of FA 20 mortars under water curing at 80 °C is still slightly increased with the increase of curing time from 21 to 28 day. According to Vasquez et al. [46], curing at elevated temperatures can better reactivate the crystalline phases of SCMs. Yang et al. [45] further indicated that curing at elevated temperatures is beneficial to the hydration of FA. Therefore, since FA has a low degree of hydration at the early age, it can still continuously hydrate under long-term water curing at 80 °C, which promotes the strength growth of the mortars.

In addition, different from water curing at 80 °C, the compressive strength of FA 20 mortars under alkali curing at 80 °C is always higher than that of GP 2 mortars. Taking alkali-activated concrete as a reference, Rakhimova and Rakhimov [47] demonstrated that alkali-activated slag cement usually requires a much lower alkali activator concentration (2–8%) than FA and metakaolin-based alkali-activated cement (5–20%). Long et al. [48] further indicated that GP can be effectively activated by using the alkali activator concentration of only 4%. In other words, like curing at elevated temperatures, a highly alkaline environment is also an effective method to improve the compressive strength of FA 20 mortars. Therefore, the higher strength of FA 20 mortars can be attributed to the promotion of external NaOH solution on the hydration/activation of FA.

## 4. Conclusions

In this study, the effects of water curing at 80 °C, the GP addition, and the GP size on the volume stability and compressive strength of GA mortars are investigated. Several new findings are drawn as follows:

(1) Compared with alkali curing at 80 °C, the volume change of GA mortars under water curing at 80 °C is negligible. On the other hand, the compressive strength of GA mortars after the 1-day water curing at 80 °C is comparable with that after the 28-day water curing at 20 °C. Therefore, the 1-day water curing at 80 °C can be used as an accelerated curing method for GA mortars. In addition, when the w/b ratio increases from 0.39 to 0.47, the ASR expansion of GA mortars increases and then decreases.
(2) With the addition of 20 wt.% GP having the mean diameter of 47.9 and 28.3 μm, the ASR expansion of GA mortars can be effectively mitigated. Furthermore, under water curing at 80 °C, the addition of GP (47.9 μm) can always obtain the higher compressive strength. It is attributed to the fact that the addition of GP (28.3 μm) cannot endow mortars with a denser particle packing state because it has a similar particle size to OPC. Therefore, the GP (47.9 μm) is suggested as an alternative material to mitigate the ASR expansion of GA mortars.
(3) The addition of FA and GP (47.9 μm) obtains the same effects on the ASR expansion of GA mortars. Moreover, after the 1-day water curing at 80 °C, the addition of GP (47.9 μm) can gain the higher compressive strength. Although the high-temperature curing and the alkaline environment are beneficial to the FA hydration, the addition of GP (47.9 μm) is still a better choice when applying the 1-day water curing at 80 °C as the accelerated curing method.

In the future, combined with the addition of GP (47.9 μm) and 1-day water curing at 80 °C, GA mortar can be considered to prepare GA concrete and be further applied to prefabricated components, because it has not only satisfactory volume stability but also excellent compressive strength.

## Figures and Tables

**Table 1:** Mixing proportions of different mixtures.

	Notation	OPC	GP	FA	GA	Water	Consistency (mm)
Series 1	Control 0.41	1	–	–	2	0.41	97 ± 2
	GP 0.5/GP 2/GP 4	0.8	0.2	–	2	0.41	101 ± 2/95 ± 1/93 ± 2
	FA 20	0.8	–	0.2	2	0.41	104 ± 3
Series 2	Control 0.41	1	–	–	2	0.41	97 ± 2
	Control 0.39	1	–	–	2	0.39	93 ± 1
	Control 0.47	1	–	–	2	0.47	107 ± 2

## Data Availability

Not applicable.
